# Untargeted Metabolomic Profiling, Multivariate Analysis and Biological Evaluation of the True Mangrove (*Rhizophora mucronata* Lam.)

**DOI:** 10.3390/antiox8100489

**Published:** 2019-10-16

**Authors:** Nabeelah Bibi Sadeer, Gabriele Rocchetti, Biancamaria Senizza, Domenico Montesano, Gokhan Zengin, Ahmet Uysal, Rajesh Jeewon, Luigi Lucini, Mohamad Fawzi Mahomoodally

**Affiliations:** 1 Department of Health Sciences, Faculty of Science, University of Mauritius, Réduit 80837, Mauritius; nabeelah.sadeer1@umail.uom.ac.mu (N.B.S.); r.jeewon@uom.ac.mu (R.J.); 2 Department for Sustainable Food Process, Università Cattolica del Sacro Cuore, Via Emilia Parmense 84, 29122 Piacenza, Italy; Gabriele.Rocchetti@unicatt.it (G.R.); biancam.senizza@virgilio.it (B.S.); 3 Department of Pharmaceutical Sciences, Food Science and Nutrition Section, University of Perugia, Via S. Costanzo 1, 06126 Perugia, Italy; 4 Department of Biology, Science Faculty, Selcuk University, Campus, 42130 Konya, Turkey; gokhanzengin@selcuk.edu.tr; 5 Department of Medicinal Laboratory, Vocational School of Health Services, Selcuk University, 42130 Konya, Turkey; ahuysal@selcuk.edu.tr

**Keywords:** true mangrove, antimicrobial, antioxidant, phytochemicals, hyperpigmentation, oxidative stress, enzyme inhibitors

## Abstract

Currently, there is a renewed interest towards the development of plant-based pharmacophores. In this work, 16 extracts prepared from the leaves, twigs, roots and fruits of a hydro-halophyte, *Rhizophora mucronata* Lam. (Family: Rhizophoraceae), were studied for possible antioxidant activity and the phenolic profiles established. Thereafter, enzymatic inhibitory activities (α-amylase, α-glucosidase, tyrosinase, acetyl- (AChE), butyrylcholinesterase (BChE), lipase, and elastase) were assessed. The total phenolic, flavonoid, phenolic acid, tannin, flavanol and triterpenoid content were estimated using standard assays. An untargeted metabolomics-based approach, based on ultra-high-pressure liquid chromatography coupled to quadrupole-time-of-flight mass spectrometry (UHPLC-QTOF-MS) followed by multivariate statistics, was then used to comprehensively profile and describe the phenolics present. UHPLC-QTOF-MS allowed for putatively annotating 104 phenolic acids, 103 flavonols, 94 flavones, 71 anthocyanins, 66 tyrosols, 29 lignans, 15 alkylphenols and 10 stilbenes in the extracts. Nine strains (*Escherichia coli, Pseudomonas aeruginosa, Klebsiella pneumoniae*, Methicillin-resistant *Staphylococcus aureus* (MRSA)*, Salmonella enteritidis, Sarcina lutea, Proteus mirabilis, Bacillus cereus* and *Candida albicans*) were then used to investigate the antimicrobial properties. The methanolic twig extract exhibited significant reducing potential towards Cu (II)/Cu (I) and Fe (III)/Fe (II) (1336.88 ± 15.70 and 710.18 ± 21.04 mg TE/g, respectively) and was the most potent DPPH radical scavenger (807.07 ± 6.83 mg TE/g). Additionally, the methanolic twig extract showed significant inhibition against most targeted enzymes. Anti-microbial results showed that all extracts were active against MRSA. Multivariate analysis demonstrated that the phenolic profile of ethyl acetate extracts and leaves were the two most discriminative parameters in terms of solvents and organs, respectively. The present findings indicated that *R. mucronata* may be further explored for the management/prevention of oxidative stress, neurodegenerative complications and hyperpigmentation.

## 1. Introduction

The tropics and sub-tropics dwell an important ecosystem in the realm where the land meets the ocean but is sometimes overlooked. It is the mangrove forests, which shield the coastlines of several tropical countries including Mauritius [1]. Apart from their ecological importance, these plants hold another facet, which is undisclosed to the scientific community. It is the therapeutic values possessed by the plants. Thus, in this study we aimed to unveil the biological activities and phytochemical composition of an untapped mangrove species, namely *Rhizophora mucronata* Lam. Mangrove plant is used as both food and medicine when considering roots, stems, leaves, flowers and fruits. Utilization is related to the content of several nutrients, such as proteins, fats, sugars, vitamins and minerals [2]. In addition, this species presents an ideal candidate to study for several reasons: it is the most dominant mangrove species in many countries, including Mauritius; its roots are locally used against diabetes, to date no attempt has been made to validate its pharmacological propensities and an absence of detailed characterization is noticed in existing literature which markedly limits our understanding on the pharmacological features of this plant. Before the era of modern medicines, *R. mucronata* was traditionally used to manage a wide spectrum of ailments, namely angina, dysentery, haematuria, ulcers, haemorrhage, diarrhoea, nausea, fever, diabetes, hypertension, constipation, menstruation disorders, and leprosy, among others [3,4,5,6].

Nowadays, contemporary science might quibble these afore-mentioned traditional uses since their pharmaceutical effects, toxicity, efficacy and safety have not been fully unravelled yet. Only few studies have reported its biological activities, namely antioxidant, anti-inflammatory [7], antimicrobial [8], anti-diabetic [9,10] and anti-viral [11]. For example, Banerjee et al. revealed that the methanolic leaves and root extracts are important sources of antioxidant compounds [12]. Another study assessed the antidiabetic activities of the fruit extract using *in vivo* models. Extract dosage of 500–2000 mg/day/head were administered in mice for 18 days. Results were positive showing a decrease in blood glucose level [2]. The analgesic activity of *R. mucronata* was also determined in mice. Results showed that the chloroform leaves extract exhibited significant activity compared to the other extracts (water and methanolic) [13]. Furthermore, our recent comprehensive review on mangroves showed that *R. mucronata* is abounded with phytochemicals, namely triterpenoids, condensed tannins, lipids, inositol, alkaloids, and gibberellins among others [14].

**Figure 1:**
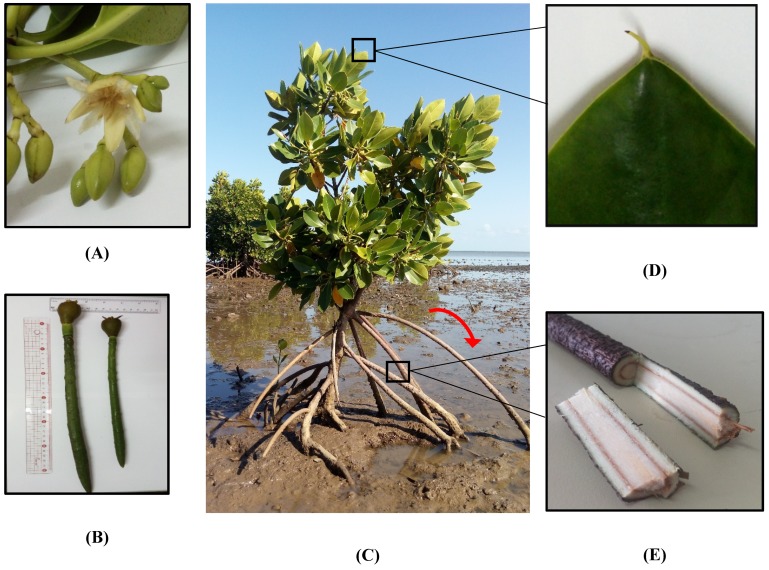
(**A**) flower, (**B**) cigar-shaped propagules, (**C**) *R. mucronata* along the coastline of Mauritius Island, (**D**) mucronate at tip of leaf, (**E**) longitudinal section of root.

In terms of morphological characteristics, *R. mucronata* is readily distinguishable by its root system since the plant has rhizophore type of root or buttress roots which grow downwards from the stem to the ground, helping the plant to be deeply rooted to the earth [3] as shown by a red arrow in Figure 1. The plant can reach a height of 3–4 m with thick leaves, dark green in colour, covered with minute black spots on the inferior surface, elliptical in shape with a mucronate at the tip. *Rhizophora mucronata* blooms creamy-white flowers and produced cigar-shaped fruits (propagules). Figure 1 illustrates the morphology of *R. mucronata*.

So far, there have been only fragmented studies on *R. mucronata,* and no in-depth investigations on the pharmacological studies have been conducted yet. This study, considered as second-to-none, is presented in four-fold: (1) to estimate the phytochemicals quantitatively using standard *in vitro* chemical tests and high-resolution mass spectrometry (i.e., ultra-high-pressure liquid chromatography coupled to quadrupole-time-of-flight mass spectrometry (UHPLC-QTOF-MS)), (2) to assess *in vitro* antioxidant capacities in terms of radical scavenging, reducing potential, total antioxidant activity and metal chelating, (3) to investigate anti-diabetic, anti-tyrosinase, anti-elastase, anti-cholinesterase activities and anti-microbial properties using nine microbial strains, and (4) to analyse the collected data using multivariate statistical approach.

## 2. Materials and Methods

### 2.1. Collection of Plant Material

Leaves, twigs, roots and fruits of *R. mucronata* were collected along the coastline of Bras D’Eau (GPS: 20°8′35.16″ S; 57°44′36.97″ E) on 25 February 2018. The plant bearing a reference number MAU 0025810 was authenticated by the botanist of the Mauritius Herbarium at the Mauritius Sugarcane Industry and Research Institute (MSIRI), Réduit, Mauritius. For sample identification, the following codes were used: RLM (*Rhizophora* leaf methanolic), RRM (*Rhizophora* root methanolic), RTM (*Rhizophora* twig methanolic), RFM (*Rhizophora* fruit methanolic), RLD (*Rhizophora* leaf decoction), RRD (*Rhizophora* root decoction), RTD (*Rhizophora* twig decoction), RFD (*Rhizophora* fruit decoction), RLA (*Rhizophora* leaf aqueous), RRA (*Rhizophora* root aqueous), RTA (*Rhizophora* twig aqueous), RFA (*Rhizophora* fruit aqueous), RLE (*Rhizophora* leaf ethyl acetate), RRE (*Rhizophora* root ethyl acetate), RTE (*Rhizophora* twig ethyl acetate), and RFE (*Rhizophora* fruit ethyl acetate).

### 2.2. Extraction

The plant parts were washed under running tap water to remove surface debris and sand and shade dried. After a constant mass was recorded, the dried plant materials were then pulverized and the finely powdered samples (50 g) were exhaustively macerated in 500 mL of three different solvents, namely methanol, ethyl acetate and distilled water.

Decoctions of each plant materials were also prepared using the following method. Briefly, 50 g of pulverized plant samples were boiled into 200 mL distilled water for 30 min. The extracts were filtered and concentrated in vacuo at 37 °C. The concentrated extracts were lyophilized and stored at +4 °C in the dark until further analysis.

### 2.3. Profile of Bioactive Compounds

With reference to our previous studies [15,16,17], total bioactive compounds, namely total phenolic (TPC), flavonoid (TFC), phenolic acid (TPA), flavanol (TFlavC), condensed tannins (TTC), and triterpenoids (TTriC) were detected by colorimetric methods. The results were expressed as mg of standard compounds (gallic acid for TPC; rutin for TFC; caffeic acid for TPA; catechin for TFlavC and TTC; oleanolic acid for TTriC) per g of dried extract.

The untargeted phenolic profile of the different *R. mucronata* extracts was investigated by means of ultra-high-pressure liquid chromatography coupled to quadrupole-time-of-flight mass spectrometer (both from Agilent Technologies, Santa Clara, CA, USA) (UHPLC-QTOF-MS). In particular, an Agilent 1290 HPLC liquid chromatography coupled to an Agilent 6550 iFunnel quadrupole-time-of-flight mass spectrometer (UHPLC/QTOF) was used. The experimental conditions for the analysis of plant extracts by means of untargeted metabolomics were optimized in our previous works [18,19]. Briefly, the mass spectrometer was run in the positive scan mode (50–1200 *m*/*z*) and chromatographic separation was achieved on an Agilent Zorbax eclipse plus C18 column (50 × 2.1 mm i.d., 1.8 μm dp) (Santa Clara, CA, USA). The LC mobile phase A consisted of water while mobile phase B was acetonitrile (LCMS grade, VWR International Ltd., Milan, Italy). The comprehensive phenolics database exported from Phenol-Explorer (http://phenol-explorer.eu/) was used for identification; with this purpose, the whole isotopic pattern (monoisotopic accurate mass, isotopic ratio and isotopic accurate spacing) was considered. This approach allowed gaining a higher confidence in the annotation step and was in compliance to the Level 2 of identification (i.e., putatively annotated compounds). The Agilent Profinder B.06 software (from Agilent Technologies, Santa Clara, CA, USA) was used also for the post-acquisition data filtering: only those compounds putatively annotated within 100% of replications in at least one condition were retained. To achieve a data reduction from the complexity of compounds annotated, as well as to provide more quantitative information, phenolic compounds were firstly ascribed into classes and subclasses, and then quantified using methanolic standard solutions (80/20, v/v methanol/water) of pure phenolic standards (purity >98%; from Sigma-Aldrich, St. Louis, MO, USA) analysed through the same method. In this regard, calibration curves were built using a linear fitting (unweighted and not forced to the axis-origin) in the range of 0.05–500 ppm, using a coefficient of determination *R^2^* > 0.98 as acceptability threshold for calibration purposes [19]. The phenolic standards used were: cyanidin (anthocyanins), catechin (flavanols and flavonols), luteolin (flavones), ferulic acid (phenolic acids), sesamin (lignans), 5-pentadecylresorcinol (alkylphenols), resveratrol (stilbenes) and tyrosol (other remaining phenolics). These compounds were considered representative of their main phenolic group. Results were finally expressed as mg phenolic equivalents/g of dried extract.

### 2.4. Determination of Antioxidant and Enzyme Inhibitory Effects

For the comprehensive insights in bio-potential of obtained extracts, their bioactivities including antioxidant, anti-α-amylase, anti-α-glucosidase, anti-cholinesterases, anti-tyrosinase, anti-lipase and anti-elastase activities assays were performed. All procedures are described in our previous papers [20,21,22]. All results were expressed as equivalents of standard compounds. These compounds were galantamine (GALAE, for cholinesterase), kojic acid (KAE, for tyrosinase), acarbose (ACAE, for amylase and glucosidase), orlistat (OE, for lipase), catechin (CAE, for elastase), Trolox (TE, for 2,2’-azino-bis(3-ethylbenzothiazoline-6-sulphonic acid (ABTS), 2,2-diphenyl-1-picrylhydrazyl (DPPH), ferric reducing antioxidant power (FRAP), cupric reducing antioxidant capacity (CUPRAC) and phosphomolybdenum, and ethylenediaminetetraacetic acid (EDTA) (EDTAE, for metal chelating).

### 2.5. Antimicrobial Properties

The microorganisms Escherichia coli ATCC 25922, Pseudomonas aeruginosa ATCC 27853, Klebsiella pneumoniae ATCC 70603, Staphylococcus aureus ATCC 43300 (MRSA), Salmonella enteritidis ATCC 13076, Sarcina lutea ATCC 9341, Proteus mirabilis ATCC 25933, Bacillus cereus ATCC 11778, Candida albicans ATCC 26555 were used for the determination of potential antimicrobial actions of R. mucronata extracts. All standard microorganisms were obtained from Microbiology Research Laboratory of Vocational School of Health Services, Selcuk University. Preparation of bacterial cultures, adjusting of McFarland density and bacterial inoculum for assays were performed according to Koc et al. [23].

The broth micro dilution method described by Zengin et al. [24] with some modifications were carried out. Extracts were initially prepared at a concentration of 25 mg/mL and added into first wells of microplates containing 100 µg/mL Mueller Hinton Broth and twofold dilutions of the extracts (6.25–0.048 mg/mL) were made by dispensing the solutions to the remaining wells. Subsequently, bacterial inoculum (100 µg/mL) was inoculated to each well then microplates were incubated at 35 °C for 18 h and *C. albicans* was incubated for two days at 28 °C. After incubation period, 2,3,5-triphenyltetrazolium chloride (TTC) (0.5%) solution was used to visualize microbial growth for the determination of minimum inhibitory concentration (MIC) values.

### 2.6. Statistical Analyses

A one-way ANOVA was performed using the software PASW Statistics 25.0 (SPSS Inc.) to investigate significant differences (*p* < 0.05, Duncan’s post hoc test) for each assay carried out. Additionally, Pearson’s correlation coefficients (*p* < 0.05) were also inspected using the same software. Thereafter, the raw metabolomic dataset exported from the Mass Profiler Professional B.12.06 (Agilent technologies) was elaborated into SIMCA 13 software (Umetrics, Malmo, Sweden) by supervised orthogonal projections to latent structures discriminant analysis (OPLS-DA) multivariate statistics. For the OPLS model, raw data were Log2 transformed, UV scaled and then analysed by OPLS-DA. The variation between the groups was separated into predictive and orthogonal (i.e., related to technical and biological variation) components. The presence of outliers into the OPLS model was checked according to Hotelling’s T2 (i.e., the distance from the origin in the model), using 95% and 99% confidence limits for suspect and strong outliers, respectively. The model cross-validation was then carried out using CV-ANOVA (*p* < 0.01), whereas permutation testing (*N* = 100) was done to exclude overfitting. Model parameters, i.e., *R^2^*Y (goodness-of-fit) and Q^2^Y (goodness-of-prediction) were also recorded. Finally, the variables’ selection method, namely variable importance in projection (VIP)), was used to evaluate the importance of each phenolic compounds in discriminating the different extraction techniques (i.e., methanol, ethyl acetate, aqueous and decoction), and to select those having the highest discrimination potential (VIP score >1).

## 3. Results and Discussion

### 3.1. Spectrophotometric Analysis of Phenolic Compounds

Polyphenols are the most abundant chemical components present in plants. It is estimated that there are about 200,000 secondary metabolites known till date which are further classified into multiple sub-classes, namely terpenes and terpenoids, alkaloids, and phenolic compounds (flavonoids, stilbenes, lignans and phenolic acids) [25,26]. Flavonoids are the most studied group of phytochemicals further divided into flavones, isoflavones, flavonols, flavanols, flavanones, and chalcones [27]. It is acknowledged that secondary metabolites are distilled into the different parts of a plant and these compounds do not directly contribute to the primary growth and development of a plant but instead help plant species survive in their respective environment [28]. Herein, the total bioactive components of the different extracts of *R. mucronata* were investigated in terms of total phenolic content (TPC), flavonoid content (TFC), phenolic acid content (TPA), flavanol content (TFlavC), tannin content (TTC) and triterpenoid content (TTriC) as presented in Table 1.

TPC was present in a significant amount in the methanolic fruit extract (220.50 ± 3.33 mg GAE/g) followed by aqueous twig extract (214.94 ± 0.96 mg GAE/g). In terms of flavonoid content, ethyl acetate leaf extract yielded the highest amount with 41.67 ± 0.38 mg RE/g. Upon comparison of the different 16 extracts screened, it is noteworthy to mention that the methanolic twig extract possessed the highest phytochemicals in terms of TPC (214.94 ± 0.96 mg GAE/g), TPA (20.73 ± 0.70 mg CE/g), TFlavC (107.69 ± 1.16 mg CAE/g), TTC (171.43 ± 2.67 mg CAE/g) and TTriC (74.86 ± 4.23 mg OAE/g). However, for TTC, there was no statistical difference between methanolic leaf and twig extracts, since both possessed high level of condensed tannin. It could be projected that the methanol solvent used in maceration extracted the highest amount of phytochemicals compared to ethyl acetate and water since it is reported that methanol is the best universal extraction solvent and extracts most polar compounds [29]. To further support this fact, a study conducted by Hardoko and co-authors [2] showed that the ethanolic fruit extract of *R. mucronata* yielded 37.35 mg GAE/g TPA [2] while herein, the methanolic fruit extract possessed 79.55 ± 0.73 mg GAE/g which represents a two-fold increase in the TPA extracted. It is important to highlight that the production of phytochemicals by plants is influenced by a number of parameters, namely geographical locations seasonal variations, environmental conditions, nutrients uptake and exposure to pollution [30]. Consequently, the type and amount of phytoconstituents are produced with respect to the plant’s environment and living conditions since secondary metabolites are only produced as part of the defensive mechanism of a plant [31]. This is further illustrated by a study conducted by Suganthy and Devi who collected leaves of *R. mucronata* in the spring season in Pichavaram, Tamil Nadu, India. Results showed that the methanolic leaf extract possessed 598.13 ± 1.85 µg GAE/mg of TPC which is higher than the results herein (methanolic leaf extract: 207.05 ± 0.88 mg GAE/g) [32]. Thus, choice of solvent might not be the sole reason for a good extraction since the amount of phytochemicals produced by the plant varies upon growing conditions as well.

**Figure 2:**
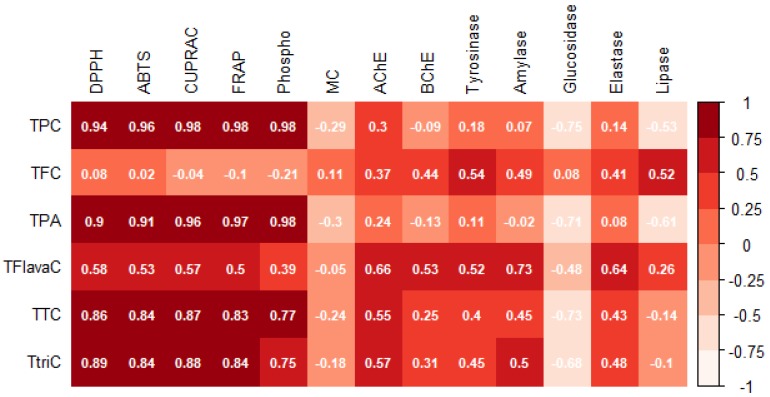
Pearson correlation plot (*p* < 0.05). Abbreviations: TPC: total phenolic content; TFC: total flavonoid content; TPA: total phenolic acid content; TFlavaC: total flavanol content; TTC: total condensed tannin content; TTriC: total triterpenoid content.

Furthermore, Pearson correlation was conducted to determine the relationship between bio compounds and biological activities. Data obtained in this study revealed strong positive correlation between TPC and DPPH (*R* = 0.94), ABTS (*R* = 0.96), CUPRAC (*R* = 0.98), FRAP (*R* = 0.98) and phosphomolybdenum (*R* = 0.98) (see Figure 2). Compared to TPC, TFC revealed lower R values in the range of −0.21 to 0.54 with all biological activities including enzymatic assays (see Figure 2).

### 3.2. Untargeted Metabolomic Profiling and Multivariate Statistics

To provide additional insights into the bioactive composition of *R. mucronata*, the phenolic composition of the methanolic, ethyl acetate, aqueous and decoction extracts was investigated with an untargeted metabolomics-based approach (based on high-resolution UHPLC-QTOF mass spectrometry). The experimental design allowed for putatively annotating 104 phenolic acids, 103 flavonols, 94 flavones, 71 anthocyanins, 66 tyrosols, 29 lignans, 15 alkylphenols and 10 stilbenes. All phenolic compounds were reported together with their composite mass spectra (Supplementary Materials). In particular, when considering the class of phenolic acids, an abundance of hydroxycinnamic and hydroxybenzoic acids like gallic acid, stigmastanol ferulate, caffeoylquinic and feruloylquinic acids were observed. Furthermore, a prevalence of glycosidic forms of procyanidin, quercetin, kaempferol and myricetin were observed among flavonoids. Additionally, apigenin, luteolin and their glycosides were the flavones most frequently detected (Supplementary Materials).

Furthermore, all polyphenols were classified and quantified using a semi-quantitative analysis according to a standard per phenolics class. The results are presented in Table 2 and expressed as mg/g dry weight. Specifically, when considering methanol as extraction solvent, leaves possessed the highest content of polyphenols being 96.9 mg/g, followed by twigs (59.34 mg/g). Interestingly, in both cases, flavanols showed the highest content, being 48.1 mg/g and 20.2 mg/g, respectively. Regarding the ethyl acetate extracts, the highest recovery of polyphenols was observed when considering tyrosols, being 18.5 mg/g in twigs, 14.8 mg/g in fruits and 9.2 mg/g in roots. Conversely, in leaves, flavonols were found to be the most abundant class, with a content of 15.0 mg/g. Similarly, concerning decoction, flavanols were better extracted in leaves (with a content of 38.6 mg/g) and twigs (recording a content of 15.8 mg/g). Considering all the different *R. mucronata* parts analysed, the polyphenol content recorded for aqueous extracts was lower when compared to the other solvents exploited, thus allowing to postulate that water was the less effective solvent in promoting the extraction of bioactive polyphenols. For the ethyl acetate extract, the highest phenolic content was found in twigs, with an abundance of 64.9 mg/g, including a majority of tyrosol equivalents (18.5 mg/g) and phenolic acids (16.4 mg/g). In addition, the lowest polyphenol content was recorded in roots (35.2 mg/g), with a scarce extraction of flavones and lignans, but an abundance of tyrosols (i.e., 9.2 mg/g). Overall, when considering the type of extracts, methanolic extracts provided better results, followed by the ethyl acetate, decoction and aqueous fraction. Interestingly, anthocyanins were found to be the phenolic sub-class better extracted in aqueous twigs. On the other hand, leaves treated with methanol allowed for obtaining the highest content of flavonols, while phenolic acids were better extracted with ethyl acetate when considering roots.

**Figure 3:**
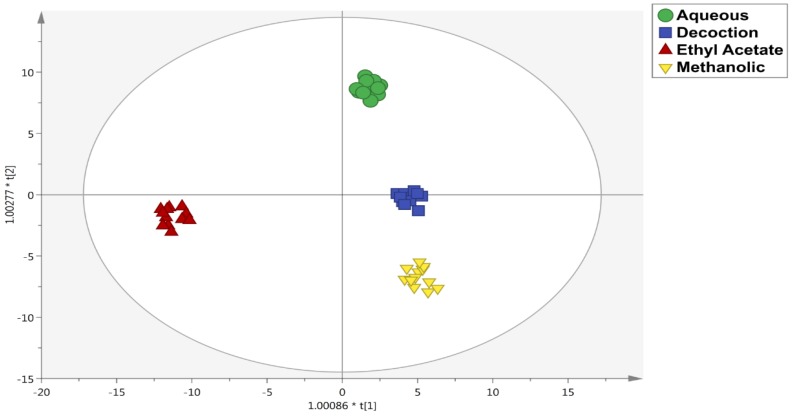
Orthogonal projections to latent structures discriminant analysis (OPLS-DA) model on extraction solvents.

**Figure 4:**
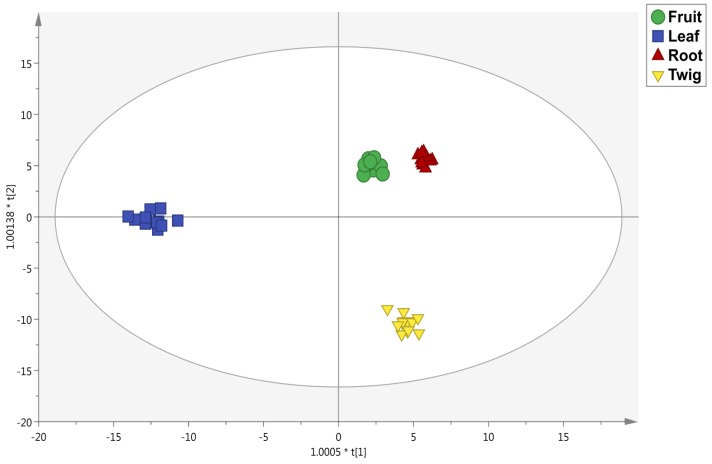
OPLS-DA model on different *Rhizophora mucronata* parts.

Afterwards, to identify the contribution of each group of phenolic compounds for discrimination purposes based on extraction solvent and matrix-type, the supervised OPLS-DA multivariate statistical approaches was carried out. The OPLS-DA score plot on solvent is reported in Figure 3. This plot underlined clear differences between methanolic, ethyl acetate, decoction and aqueous methods with statistically supported values, on the basis of the polyphenol profiles detected. Notably, the phenolic profile of ethyl acetate was clearly discriminated from the others, while decoction and aqueous samples were found to be very similar, probably due to the use of water as extraction solvent in both cases. The discriminant model parameters were characterized by acceptable values, being R^2^Y (goodness-of-fit) = 0.98 and Q^2^Y (goodness-of-prediction) = 0.94. Subsequently, the VIP selection method was applied to evaluate the variables importance in projection of the OPLS-DA model built, then identifying 59 compounds able to discriminate each extraction method used. The most important compounds are reported in Supplementary Materials with their prediction score (>1.2) standard error, highlighting the presence of 21 phenolic acids, 17 flavonoids, 6 lignans and 4 phenolic terpenes. In this regard, the most recorded phenolic acids were hydroxybenzoic and hydroxycinnamic acids. Finally, a second OPLS-DA model (Figure 4) was carried out to point out differences between the four organs of the plant under investigation. The score plot (possessing R^2^Y and Q^2^Y values of 0.99 and 0.96, respectively) showed four distinct groups, each one represented by leaves, roots, fruits and twigs. Interestingly, leaves were better discriminated than the others; in particular, the similarity in phenolics profiles between fruits and roots was clear. In addition, the VIP approach with a prediction score >1.2 allowed for identifying 71 compounds belonging to the class of flavonoids (41 compounds), phenolic acids (15 compounds) and other polyphenols (13 compounds) (Supplementary Materials). However, the selection of the extraction solvent for the four organs (i.e., fruits, leaves, roots and twigs) should be carefully evaluated in order to promote a selective extraction of bioactive compounds.

### 3.3. In Vitro Antioxidant Assays

As part of the normal bodily functions and defensive mechanisms, the body neutralized free radicals efficiently through a range of different types of antioxidants [33]. The human defence system involves both endogenous and exogenous antioxidants to prevent noxious free radicals from harming the human body. Nevertheless, enzymatic defences against certain types of reactive oxygen species (e.g., singlet oxygen and hydroxyl radical) are either ineffective or totally lacking. An imbalance between antioxidants and free radicals represents a paragon of an unhealthy functioning body and leads to numerous diseases, namely cancer, atherosclerosis, Alzheimer’s, Parkinson’s disease, gastric ulcers, and inflammation among others [34]. Thus, as long as diseases remain perennial, our quest for potent and novel drugs should never be halted. As a result, the current study attempts at screening all prepared extracts through a series of antioxidant assays, namely free radical scavenging (DPPH, ABTS), reducing power (FRAP, CUPRAC), total antioxidant capacity (phosphomolybdenum) and metal chelating.

The highest and lowest DPPH activity was recorded in the methanolic twig (807.07 ± 6.83 mg TE/g) and ethyl acetate fruit extracts (41.89 ± 1.76 mg TE/g) respectively. This result is supported by the polyphenolic composition which showed that the methanolic twig extract contained the highest TPC, particularly TPA, TFlavC, TTC and TTriC, and the ethyl acetate fruit extract yielded the lowest TPC, including TPA, TFlavC and TTC (Table 1). Thus, it can be suggested that the DPPH activity was directly linked to the phenolic compounds present. Moreover, the ability for ABTS radical scavenging was also investigated since ABTS•^−^ chromogen has numerous advantages over DPPH radical chromogen. For instance, ABTS has the ability to work with both lipophilic and hydrophilic compounds while DPPH can only be solubilized in organic media [35]. Since we have prepared both organic and aqueous extracts for *R. mucronata*, the usage of both DPPH and ABTS radicals are thus justified. Indeed, our results clearly support the fact reported by Kim et al. [35]. For instance, DPPH assay classified methanolic twig extract as most active while ABTS classified both methanolic twig and aqueous twig extract as the most potent ABTS scavengers (Table 3).

Furthermore, the antioxidant capacity of the studied extracts was evaluated in terms of reducing power using CUPRAC and FRAP assays. Several factors governed the reducing potential of antioxidants, namely their ionization potentials, spin distribution of the radical cations and the bond dissociation energy of the phenolic O-H bond [36]. With CUPRAC assay, 1 g of methanolic twig extract exhibited a remarkably high Trolox equivalent value (1336.88 mg TE) followed by aqueous twig (1082.00 mg TE). Likewise, a similar trend was noticed with FRAP assay, viz. the most potent extract being methanolic twig followed by aqueous twig. The untargeted metabolomic profile reported that the methanolic twig extract yielded relatively high level of polyphenols with 59.34 mg/g. In addition, from Table 3, it is shown that the latter extract possessed a high amount of phenolic compounds and triterpenoids. However, a low level of flavonoids was noted. Therefore, the reducing potential of the tested samples could be ascribed to the presence of phenolic and triterpenoid compounds rather than flavonoids, as a good correlation was observed between phenolic compounds and reducing power (see Figure 2). As a supportive information, Tanaka et al. [37] stated that the antioxidant capacities are concomitantly linked to the reducing power of phytochemicals.

Secondary metabolites are known to exhibit significant and substantial antioxidant properties, not only based on their ability to donate electrons but also by chelating transition metals [38]. Table 3 shows the different metal chelating activities of the extracts from different parts of *R. mucronata*. These data depict that methanolic fruit extract is the most effective metal chelator followed by aqueous fruit extract (26.36 ± 0.51 and 18.82 ± 2.48 mg EDTAE/g, respectively). Additionally, the studied samples were assessed for their total antioxidant capacity (phosphomolybdenum assay). The latter assay is based on the reduction of Mo (VI) to Mo (V) by antioxidants and as a result a green complex is formed in acidic media [39]. The aqueous twig extract showed significant antioxidant capacity (5.06 ± 0.13 mmol TE/g). Interestingly, this particular extract yielded the highest phenolic acid content (21.00 ± 1.18 mg CE/g). It could be projected that TPA was responsible for the total antioxidant capacity since the strongest Pearson correlation coefficient (*R* = 0.98) was observed between those two parameters (see Figure 2).

### 3.4. Enzymatic Inhibitory Assays

Currently, there is an alarming rise in both mortality and morbidity rate associated with non-communicable diseases (NCDs), namely cancers, diabetes, and neurodegenerative diseases, spreading across the globe, mainly affecting developing countries [40]. Thus, there is a dire need to develop novel countermeasures for such diseases and to improve the existing drugs to effectively manage diseases. In the present study, the different extracts of *R. mucronata* were evaluated against key enzymes, namely α-amylase, α-glucosidase, tyrosinase, elastase, and lipase, acetyl- and butyryl-cholinesterase (AChE and BChE, respectively). The results are shown in Table 4.

Inhibition of gut enzymes, like α-amylase and α-glucosidase have been suggested to be beneficial in the management of diabetes mellitus type II (DMII), particularly to reduce postprandial hyperglycemia α-Amylase acts on dietary polysaccharides such as starch to form disaccharides (maltose) which are subsequently broken further into monosaccharides (glucose) by α-glucosidase [41]. Natural inhibitors of such enzymes, particularly from plant origin are currently high on the research agenda geared towards the discovery of novel safe antidiabetic drugs. Scrutinizing and promoting *R. mucronata* as a future antidiabetic phytomedicine reveals to be a promising approach considering the fact that a decoction of the root of the plant is believed to manage diabetes in folklore medicine. Results showed that the different studied samples were good inhibitors of α-amylase. The highest activity was recorded with the methanolic leaf and methanolic twig extracts (0.96 ± 0.03 and 0.95 ± 0.01 mmol ACAE/g, respectively). However, it was noted that these extracts were not active against α-glucosidase. In fact, all methanolic extracts were found to be inactive against α-glucosidase in contrast to α-amylase. Instead, both aqueous root and aqueous fruit extracts showed highest activity against α-glucosidase (31.16 ± 0.28 and 31.16 ± 0.04 mg ACAE/g, respectively). Bearing in mind that a decoction of the root of *R. mucronata* are locally used in Mauritius to manage diabetes, however, after this pharmacological validation, it can be said that the results offered only a partial support to the traditional uses of the mangrove plant since the decocted root was not the most potent inhibitor against α-amylase and α-glucosidase.

Obesity is recognised as a chronic and non-communicable disease related to unwanted weight gain due to excess fat accumulation [42]. Obesity is the hallmark disease of a range of health problems, namely diabetes, cancer, cardiovascular diseases [43]. Irrespective of the causes, obesity and weight gain have quickly mushroomed over the past few years threatening the lives of millions of people globally. As ample evidence, the World Health Organization (WHO) in 2016 has recorded about 650 million obese people (>18 years old) [43]. The two most influential anti-obesity drugs are namely Orlistat ™ (Xenical) and Sibutramine (Meridia). However, the latter drug carries some serious side effects linked to the nervous system of patients [44,45]. Thus, searching for safer anti-obesity drugs remain pertinent. It is noteworthy to point out that obesity and diabetes are inter-related. This is supported by a study conducted by Ramirez et al. [46] demonstrating that an alarming increase of postprandial glucose and free fatty acids reaching supra-physiological levels may lead to β-cell failure resulting in the suppression of insulin formation which results in high blood glucose level. Since traditional knowledge claimed that *R. mucronata* can alleviate DMII, we opted to scrutinize our extracts against pancreatic lipase enzyme as well. To properly evaluate anti-obesity agents, it is recommended to determine their pancreatic lipase activity [47]. Data collected in this study showed that the ethyl acetate fruit extract depressed pancreatic lipase activity the most (101.02 ± 1.31 mg OE/g) followed by the ethyl acetate root extract (88.32 ± 2.18 mg OE/g). It is noted that not all extracts exhibited lipase activity (Table 3). Although it is reported that flavonoids can play an important role in the management of obesity [41], the results presented herein are not in agreement with this fact since the extracts aforementioned do not possess the highest flavonoid content (TFC) (Table 2).

Other commonly known diseases are the neurodegenerative diseases, namely Alzheimer’s and Parkinson’s disease. Neurodegenerative ailments enclose a group of heterogeneous diseases that are triggered by a gradual deterioration of the function of the central or peripheral nervous system [48]. Cholinesterase inhibitors increase level of acetylcholine in cholinergic synapses which consequently facilitates neurotransmission [49]. In this work, all samples exhibited activity against AChE; however, the aqueous root and aqueous fruit were inactive against BChE. Interestingly, all four methanolic extracts (leaf, root, twig and fruit) were the most effective inhibitors against AChE (4.60 ± 0.02, 4.61 ± 0.05, 4.78 ± 0.03 and 4.46 ± 0.18 mg GALAE/g respectively). On the other hand, different extracts showed high activity against BChE, namely methanolic root, methanolic twig and ethyl acetate fruit, and no statistical difference were recorded between them (Table 4). Although flavonoids are reported to be efficient cholinesterase inhibitors, results from this study do not correlate with the observed cholinesterase activities [50]. For instance, the ethyl acetate leaf extract which yielded the highest flavonoid content did not show high cholinesterase activity.

Apart from the pharmaceutical world, the cosmeceutical industry is one of the fastest growing components of the natural care industry. The usage of cosmetics in our daily life is not recent but dates back to 6000 years ago. Cosmetics and skin care products are woven into our everyday grooming. Dorni et al. [51] stated that plants are the primary sources of phytochemicals having the potential to rejuvenate human skins. The two most important cosmetic enzymes are tyrosinase and elastase. In the continuous quest for products of cosmetic importance, we screened our samples against these enzymes. Melanin is a pigment that determines skin colour. This pigment is secreted and produced by the melanocyte cells through a physiological process called melanogenesis. The key enzyme that is responsible for melanin production is tyrosinase. An excessive production of melanin results in skin disorders, namely hyperpigmentation and melanoma [52,53]. Besides hyperpigmentation issues, facial wrinkles and sagging are another dermatological problem feared by many people, especially women. Skin ageing is fuelled by chronic exposure to ultraviolet radiation leading to the formation of reactive oxygen species (ROS) resulting in the loss of skin elasticity which subsequently becomes the cause of wrinkle formation, sagging, brown spots, skin cancer, melanoma among others. The major contributor behind the formation of wrinkles and dehydration of skin is elastase enzyme [54]. The current study achieved promising tyrosinase and elastase inhibitory results. In fact, according to statistical analysis, the highest anti-tyrosinase activities were observed with methanolic twig and methanolic leaf extracts (145.31 ± 1.49 and 144.02 ± 0.74 mg KAE/g, respectively) while methanolic leaf, root, twig together with ethyl acetate fruit extracts were the most potent elastase inhibitors (4.58 ± 0.04, 4.50 ± 0.16, 4.68 ± 0.08, 4.25 ± 0.25 mg CAE/g, respectively) (Table 4).

### 3.5. Antimicrobial Properties

In this study antimicrobial potentials of *R. mucronata* extracts were evaluated by micro broth dilution method. The minimum inhibitory concentrations of extracts are given in Table 5. When the methanol extracts of *R. mucronata* were assessed, leaf methanol extract revealed significant antimicrobial activity against methicillin-resistant *Staphylococcus aureus* (MRSA) at a dose of 0.19 mg/mL (Table 5). The same extract was effective against *S. enteritidis* and *S. lutea* at a concentration of 1.56 mg/mL, while the MIC value of this extract was determined as 0.39 µg/mL against *P. mirabilis*. The methanolic root extract exhibited moderate antimicrobial activity only against MRSA with 0.39 mg/mL MIC value. Except for MRSA, other bacteria were resistant to this extract. The methanolic twig extract showed moderate antimicrobial activity at doses ranging between 1.56–0.39 mg/mL. *P. mirabilis* and MRSA were the most sensitive bacteria against this extract with 0.39 mg/mL MIC values, while the MIC values were determined as 1.56 mg/mL against *E. coli, S. enteritidis* and *S. lutea*. The methanolic fruit extract was only effective against *E. coli* and MRSA, but *E. coli* was more sensitive than MRSA with 1.56 mg/mL MIC value. *Pseudomonas aeruginosa*, *B. cereus* and *C. albicans* were resistant to all methanol extracts (Table 5).

The *R. mucronata* leaf decoction showed weak to moderate antimicrobial activities against strains tested. MRSA strain was the most sensitive bacterium with 0.39 mg/mL MIC value (Table 5). *Proteus. mirabilis* was the second sensitive bacterium to this extract with 1.56 mg/mL dose, while *E. coli*, *P. aeruginosa* and *B. cereus* affected from this extract at a concentration of 6.25 mg/mL. The root decoction was only effective against MRSA and *P. mirabilis* with 0.78 and 6.25 mg/mL MIC values, respectively. Twig decoction manifested MIC values ranging between 6.25–0.39 mg/mL and MRSA affected from this extract at a dose of 0.39 mg/mL so this strain was assessed as the most sensitive bacterium. Fruit decoction was determined as weak antimicrobial by 6.25 µg/mL MIC values against *E. coli* and MRSA. As methanol extracts, the decoctions of *R. mucronata* parts have no antifungal activity against *C. albicans*. However, leaf and twig decoctions have weak antimicrobial activities against *P. aeruginosa* (Table 5).

The leaf aqueous extract was effective on MRSA bacterium with 0.39 mg/mL MIC value (Table 5). It revealed weak antifungal activity against *C. albicans* at a dose of 6.25 mg/mL. The root aqueous extract was effective against MRSA and *P. mirabilis* at concentrations of 1.56 and 6.25 mg/mL, respectively. The twig aqueous extract manifested antimicrobial reaction against all tested bacteria with MIC values ranging between 6.25–0.39 mg/mL. Also, it has antifungal capacity. MRSA was the most sensitive bacterium against twig aqueous extract followed by *P. mirabilis*. MIC value was determined as 3.12 mg/mL against *B. cereus* which is resistant to other extracts except for leaf decoction.

Excluding MRSA strain, the ethyl acetate extracts of *R. mucronata* were not effective against the tested microorganisms. MIC values were determined as 1.56 and 3.12 mg/mL for this bacterium. Overall it can be stated from this study that all extracts were effective on MRSA strains and *R. mucronata* leaf methanol was the most effective extract with 0.19 mg/mL MIC value. Also twig and leaf aqueous extracts have weak antifungal capacity against *Candida.*

Methicillin-resistant *Staphylococcus aureus* (MRSA) is an important nosocomial and community-acquired pathogen that has also developed resistance to various antibiotics (β-lactams, quinolones, and aminoglycosides) [55]. MRSA infections cause a large number of deaths every year worldwide [56]. Vancomycin was considered to be the last-resort antibiotic for the treatment of MRSA infections, but MRSA resistance to vancomycin has been reported too [57,58]. This suggests that MRSA will likely acquire more resistance to vancomycin in the near future. Therefore, it is increasingly necessary to discover new antibiotics or to devise new measures that are effective against MRSA infections. Our data showed that there was no uniform response within or between the bacterial strains in terms of susceptibility to methanol, decoction, aqueous and ethyl acetate extracts of *R. mucronata* parts. It was determined that some extracts of the plant had significant antibacterial and anti-MRSA activities and they may be used in the treatment of the MRSA infections.

## 4. Conclusions

*Rhizophora mucronata* is a currently underexplored mangrove species, both in terms of its biopharmaceutical potential and phytochemical composition. Data collected from this study highlighted that this mangrove plant exhibited pronounced antioxidant and enzyme-inhibitory activities. In our experimental condition, the methanolic twig extract showed the highest yields for TPA (20.73 mg CE/g), TTriC (74.86 mg OAE/g), TTC (171.43 mg CAE/g), TPC (220.50 mg GAE/g), and displayed substantial FRAP reducing power (710.18 mg TE/g). On the other hand, the aqueous and methanolic leaf extracts were characterized by an averaged ABTS value of 602.91 mg TE/g. Interestingly, we found that methanolic extracts did not show α-glucosidase inhibition when compared to the other extracts tested, while ethyl acetate fruit extract possessed lipase inhibition activity of 101.02mg OE/g. Therefore, the presence of phenolic compounds in the methanolic extracts of *R. mucronata* represent potential skin protectors against ROS and thus could prevent skin ageing and wrinkles. After pharmacological validation, it can be said that the results offer partial support to the traditional uses of the mangrove plant since the decocted root was not the most potent inhibitor against α-amylase, α-glucosidase and pancreatic lipase enzymes. Nonetheless, the actual phenolic profile was strongly affected by the extraction solvent, with organic solvents (methanol and ethyl acetate) showing marked differences versus water extracts. Enzyme inhibition assays provided indications on the potential therapeutic value of *R. mucronata* for the management of epidermal hyperpigmentation and neurodegenerative complications. However, further research including clinical *in vivo* studies is recommended to further evaluate these aforementioned properties, in order to include this traditional plant as a potential cosmetic, nutraceutical and pharmacological ingredients.

## Figures and Tables

**Table 1:** Extraction yields (%) and total bioactive components of *R. mucronata* extracts.

Samples	Yield (%)	Total Phenolic Content (mg GAE/g)	Total Flavonoid Content (mg RE/g)	Total Phenolic Acid Content (mg CE/g)	Total Flavanol Content (mg CAE/g)	Total Condensed Tannin Content (mg CAE/g)	Total Triterpenoid Content (mg OAE/g)
**RLM**	26.88	207.05 ± 0.88 ^c^	31.85 ± 0.31 ^b^	14.59 ± 0.89 ^d^	98.77 ± 0.92 ^b^	173.69 ± 8.36 ^a^	64.40 ± 4.10 ^b^
**RRM**	20.90	107.98 ± 1.49 ^h^	2.59 ± 0.09 ^j,k^	7.89 ± 0.21 ^g^	52.31 ± 0.56 ^c^	93.99 ± 0.77 ^b^	26.72 ± 2.49 ^e^
**RTM**	14.38	220.50 ± 3.33 ^a^	12.46 ± 0.19 ^g^	20.73 ± 0.70 ^a^	107.69 ± 1.16 ^a^	171.43 ± 2.67 ^a^	74.86 ± 4.23 ^a^
**RFM**	6.94	79.55 ± 0.73 ^j^	2.69 ± 0.11 ^j^	5.55 ± 0.09 ^i^	21.25 ± 0.28 ^e^	43.75 ± 2.68 ^f^	13.78 ± 0.47 ^g^
**RLD**	21.62	173.89 ± 1.43 ^f^	19.26 ± 0.15 ^d^	17.63 ± 0.30 ^c^	12.24 ± 0.08 ^g,h^	73.52 ± 1.60 ^d^	31.26 ± 2.01 ^c,d^
**RRD**	37.14	104.54 ± 2.68 ^h^	2.17 ± 0.07 ^j,k,l^	9.40 ± 0.37 ^f^	3.86 ± 0.03 ^i^	38.78 ± 1.56 ^f^	14.22 ± 0.41 ^g^
**RTD**	15.08	188.55 ± 0.89 ^d^	4.08 ± 0.16 ^i^	19.14 ± 0.93 ^b^	12.75 ± 0.13 ^g^	80.69 ± 7.83 ^c^	34.14 ± 1.00 ^c,d^
**RFD**	20.44	57.29 ± 0.32 ^k^	1.79 ± 0.05 ^l^	3.80 ± 0.07 ^j^	2.07 ± 0.02 ^j^	19.33 ± 0.39 ^h,i^	4.81 ± 0.52 ^i^
RLA	29.78	178.15 ± 0.83 ^e^	6.00 ± 0.23 ^h^	14.27 ± 0.98 ^d^	12.80 ± 0.04 ^g^	95.56 ± 4.67 ^b^	30.15 ± 2.32 ^d,e^
**RRA**	11.54	124.02 ± 1.09 ^g^	1.65 ± 0.44 ^l^	11.43 ± 0.43 ^e^	4.73 ± 0.08 ^i^	57.44 ± 2.06 ^e^	19.00 ± 1.62 ^f^
**RTA**	8.82	214.94 ± 0.96 ^b^	4.09 ± 0.22 ^i^	21.00 ± 1.18 ^a^	14.93 ± 0.22 ^f^	93.64 ± 2.29 ^b^	35.12 ± 3.09 ^c^
**RFA**	3.24	96.57 ± 0.44 ^i^	1.87 ± 0.24 ^k,l^	7.12 ± 0.19 ^g,h^	2.69 ± 0.06 ^j^	25.69 ± 0.56 ^g,h^	9.33 ± 0.97 ^h^
**RLE**	8.06	41.83 ± 1.27 ^m^	41.67 ± 0.38 ^a^	1.79 ± 0.05 ^k^	11.45 ± 0.15 ^h^	16.94 ± 0.58 ^i^	10.05 ± 0.49 ^g,h^
**RRE**	4.54	51.14 ± 0.69 ^l^	14.78 ± 0.30 ^e^	3.02 ± 0.36 ^j^	4.16 ± 0.05 ^i^	30.40 ± 1.72 ^g^	11.97 ± 0.45 ^g,h^
**RTE**	1.32	97.13 ± 4.16 ^i^	13.39 ± 0.18 ^f^	6.23 ± 0.24 ^h,i^	49.73 ± 1.19 ^d^	57.03 ± 0.95 ^e^	22.33 ± 1.52 ^f^
**RFE**	0.76	31.06 ± 0.05 ^n^	24.36 ± 1.13 ^c^	1.64 ± 0.10 ^k^	2.65 ± 0.01 ^j^	7.95 ± 0.74 ^j^	10.60 ± 1.66 ^g,h^

Different letters(^a–n^) indicate significant differences in the tested extracts (*p* < 0.05). Values are expressed as mean ± S.D. of three parallel measurements. Abbreviations: RLM: *Rhizophora* leaf methanolic, RRM: *Rhizophora* root methanolic, RTM: *Rhizophora* twig methanolic, RFM: *Rhizophora* fruit methanolic, RLD: *Rhizophora* leaf decoction, RRD: *Rhizophora* root decoction, RTD: *Rhizophora* twig decoction, RFD: *Rhizophora* fruit decoction, RLA: *Rhizophora* leaf aqueous RRA: *Rhizophora* root aqueous, RTA: *Rhizophora* twig aqueous, RFA: *Rhizophora* fruit aqueous, RLE: *Rhizophora* leaf ethyl acetate RRE: *Rhizophora* root ethyl acetate, RTE: *Rhizophora* twig ethyl acetate, RFE: *Rhizophora* fruit ethyl acetate; GAE: Gallic acid equivalent; RE: Rutin equivalent; CE: Caffeic acid equivalent; CAE: Catechin equivalent; OAE: Oleanolic acid equivalent.

**Table 2:** Semi-quantification of the major phenolic sub-classes according to standard equivalent compounds.

Samples	Cyanidin Eq.	Luteolin Eq.	Catechin Eq.	Sesamin Eq.	Tyrosol Eq.	Ferulic Acid Eq.
**RLM**	11.88 ± 0.80 ^a^	4.03 ± 0.57	48.12 ± 0.7 ^a^	2.49 ± 0.23 ^a,b^	4.73 ± 0.05 ^b^	16.26 ± 0.29 ^a^
**RRM**	9.64 ± 0.19 ^a,b^	4.79 ± 1.13	12.69 ± 0.7 ^c^	1.48 ± 0.07 ^b^	5.84 ± 1.38 ^b^	14.93 ± 1.25 ^a,b^
**RTM**	6.07 ± 1.78 ^b^	4.37 ± 0.81	20.18 ± 4.2 ^b^	3.58 ± 0.17 ^a^	5.48 ± 0.87 ^b^	12.54 ± 1.66 ^b^
**RFM**	4.53 ± 0.16 ^b^	4.06 ± 2.21	8.10 ± 0.2 ^d^	1.19 ± 0.16 ^b^	6.04 ± 0.13 ^a,b^	6.69 ± 0.48 ^c^
**RLE**	9.70 ± 0.98 ^a^	4.85 ± 0.51 ^a^	15.04 ± 3.8 ^a^	2.35 ± 0.14 ^a,b^	9.44 ± 0.29 ^b^	14.18 ± 1.08 ^b^
**RRE**	6.47 ± 0.32 ^a,b^	1.81 ± 0.20 ^b^	8.88 ± 0.4 ^c^	1.29 ± 0.14 ^b^	9.21 ± 1.20 ^b^	4.31 ± 0.59 ^c^
**RTE**	10.60 ± 0.85 ^a^	2.27 ± 0.11 ^b^	11.92 ± 3.3 ^b^	3.08 ± 0.05 ^a^	18.50 ± 0.71 ^a^	16.36 ± 3.24 ^a,b^
**RFE**	4.26 ± 0.85 ^b^	1.56 ± 0.20 ^b^	6.84 ± 0.7 ^c^	1.71 ± 0.55 ^b^	14.77 ± 5.55 ^a^	7.29 ± 4.62 ^b,c^
**RLD**	9.10 ± 0.75 ^a^	7.72 ± 0.29 ^a^	38.56 ± 0.8 ^a^	1.90 ± 0.22 ^b^	3.36 ± 0.82 ^b^	5.56 ± 1.18 ^c^
**RRD**	6.37 ± 0.50 ^b^	1.73 ± 0.12 ^c^	9.41 ± 1.2 ^c^	1.53 ± 0.05 ^b^	5.16 ± 0.94 ^a^	19.24 ± 1.07 ^a^
**RTD**	7.82 ± 1.17 ^a,b^	5.37 ± 0.82 ^b^	15.79 ± 3.2 ^b^	2.89 ± 0.15 ^a,b^	4.32 ± 0.09 ^a,b^	12.88 ± 0.69 ^b^
**RFD**	5.10 ± 0.65 ^b^	2.06 ± 0.06 ^c^	10.10 ± 0.4 ^c^	2.47 ± 0.73 ^a,b^	5.86 ± 0.64 ^a^	10.82 ± 1.12 ^b^
**RLA**	7.37 ± 0.42 ^b^	4.06 ± 0.51 ^a,b^	25.72 ± 0.2 ^a^	1.93 ± 0.09 ^a,b^	6.33 ± 0.21 ^a,b^	5.75 ± 0.911 ^b^
**RRA**	7.83 ± 0.21 ^b^	2.23 ± 0.17 ^c^	16.77 ± 0.2 ^a,b^	1.40 ± 0.03 ^a,b^	6.35 ± 1.43 ^a,b^	8.39 ± 0.27 ^a^
**RTA**	12.50 ± 0.26 ^a^	3.16 ± 0.48 ^b^	12.56 ± 4.1 ^b^	0.75 ± 0.06 ^b^	5.86 ± 0.91 ^a,b^	5.42 ± 1.11 ^b^
**RFA**	4.01 ± 0.60 ^c^	1.64 ± 0.21 ^c^	6.64 ± 0.2 ^c^	2.56 ± 0.05 ^a^	4.42 ± 0.12 ^b^	6.91 ± 0.18 ^a,b^

Results are provided as mean ± standard deviation (*n* = 3) and expressed as mg equivalents (Eq.)/g dry weight. Different superscript letters (^a–c^) indicate significant (*p* < 0.05) differences in the tested different extracts (i.e., methanolic, decoction, aqueous and ethyl acetate) as resulted by Duncan’s *post-hoc* test. Abbreviations: RLM: *Rhizophora* leaf methanolic, RRM: *Rhizophora* root methanolic, RTM: *Rhizophora* twig methanolic, RFM: *Rhizophora* fruit methanolic, RLD: *Rhizophora* leaf decoction, RRD: *Rhizophora* root decoction, RTD: *Rhizophora* twig decoction, RFD: *Rhizophora* fruit decoction, RLA: *Rhizophora* leaf aqueous RRA: *Rhizophora* root aqueous, RTA: *Rhizophora* twig aqueous, RFA: *Rhizophora* fruit aqueous, RLE: *Rhizophora* leaf ethyl acetate RRE: *Rhizophora* root ethyl acetate, RTE: *Rhizophora* twig ethyl acetate, RFE: *Rhizophora* fruit ethyl acetate.

**Table 3:** Antioxidant properties of *R. mucronata* extracts.

Samples	DPPH (mg TE/g)	ABTS (mg TE/g)	Phosphomolybdenum (mmol TE/g)	Metal Chelating (mg EDTAE/g)	CUPRAC (mg TE/g)	FRAP (mg TE/g)
**RLM**	688.96 ± 24.01 ^b,^*	602.05 ± 9.43 ^a^	4.13 ± 0.29 ^d^	10.44 ± 0.45 ^e^	1050.40 ± 9.11 ^c^	552.01 ± 20.31 ^d^
**RRM**	99.52 ± 0.10 ^g^	141.53 ± 0.10 ^f^	2.61 ± 0.13 ^f^	5.57 ± 1.01 ^f^	418.54 ± 4.39 ^f^	225.22 ± 1.44 ^g^
**RTM**	807.07 ± 6.83 ^a^	514.23 ± 7.95 ^b^	4.62 ± 0.17 ^b,c^	15.63 ± 0.56 ^c,d^	1336.88 ± 15.70 ^a^	710.18 ± 21.04 ^a^
**RFM**	97.66 ± 0.21 ^g^	140.82 ± 0.08 ^f^	1.81 ± 0.09 ^g^	26.36 ± 0.51 ^a^	318.48 ± 5.10 ^i^	182.34 ± 7.89 ^i^
**RLD**	469.04 ± 7.42 ^f^	383.52 ± 9.81 ^e^	4.32 ± 0.15 ^d^	11.34 ± 0.84 ^e^	918.08 ± 8.36 ^e^	511.93 ± 12.84 ^e^
**RRD**	96.39 ± 0.38 ^g^	140.13 ± 0.11 ^f^	2.65 ± 0.04 ^f^	3.99 ± 0.79 ^f^	388.44 ± 0.23 ^g^	222.87 ± 1.13 ^g^
**RTD**	523.48 ± 6.13 ^e^	459.17 ± 22.80 ^c^	4.69 ± 0.03 ^b^	6.33 ± 0.09 ^f^	1001.52 ± 9.53 ^d^	601.21 ± 3.65 ^c^
**RFD**	73.25 ± 0.77 ^h^	107.60 ± 4.86 ^g^	1.43 ± 0.02 ^h^	15.27 ± 0.61 ^d^	194.02 ± 2.05 ^j^	130.08 ± 1.08 ^j^
**RLA**	543.33 ± 6.63 ^d^	408.02 ± 19.70 ^d^	4.39 ± 0.16 ^c,d^	11.08 ± 1.98 ^e^	918.73 ± 8.00 ^e^	511.86 ± 3.10 ^e^
**RRA**	94.73 ± 0.14 ^g^	139.66 ± 0.06 ^f^	3.25 ± 0.04^e^	4.95 ± 0.10^f^	427.54 ± 2.31 ^f^	249.57 ± 0.65 ^f^
**RTA**	656.32 ± 20.03 ^c^	602.91 ± 7.03 ^a^	5.06 ± 0.13^a^	17.92 ± 0.96^bc^	1082.00 ± 47.96 ^b^	636.79 ± 3.13 ^b^
**RFA**	92.55 ± 0.27 ^g^	139.85 ± 0.04 ^f^	2.51 ± 0.02^f^	18.82 ± 2.48^b^	314.59 ± 8.99 ^i^	200.75 ± 4.69 ^h^
**RLE**	55.41 ± 0.89 ^i^	64.44 ± 0.60 ^h^	1.14 ± 0.03^i^	14.92 ± 0.84^d^	152.48 ± 3.68 ^k,l^	71.60 ± 3.01 ^l^
**RRE**	56.93 ± 0.09 ^h,i^	70.63 ± 1.42 ^h^	1.78 ± 0.11^g^	18.16 ± 1.65^b^	175.70 ± 0.34 ^j,k^	99.09 ± 2.43 ^k^
**RTE**	94.99 ± 0.25 ^g^	140.64 ± 0.19 ^f^	2.63 ± 0.14^f^	15.35 ± 0.21^d^	357.95 ± 1.83 ^h^	197.26 ± 2.62 ^h,i^
**RFE**	41.89 ± 1.76 ^i^	36.57 ± 1.53 ^i^	1.05 ± 0.13^i^	17.33 ± 1.98^b,c,d^	142.21 ± 6.37 ^l^	62.17 ± 0.45 ^l^

TE: Trolox equivalent; EDTAE: EDTA equivalent. Different letters (^a–l^) indicate significant differences in the tested extracts (*p* < 0.05). Values are expressed as means ± S.D. of three parallel measurements. Abbreviations: RLM: *Rhizophora* leaf methanolic, RRM: *Rhizophora* root methanolic, RTM: *Rhizophora* twig methanolic, RFM: *Rhizophora* fruit methanolic, RLD: *Rhizophora* leaf decoction, RRD: *Rhizophora* root decoction, RTD: *Rhizophora* twig decoction, RFD: *Rhizophora* fruit decoction, RLA: *Rhizophora* leaf aqueous RRA: *Rhizophora* root aqueous, RTA: *Rhizophora* twig aqueous, RFA: *Rhizophora* fruit aqueous, RLE: *Rhizophora* leaf ethyl acetate RRE: *Rhizophora* root ethyl acetate, RTE: *Rhizophora* twig ethyl acetate, RFE: *Rhizophora* fruit ethyl acetate.

**Table 4:** Enzyme inhibitory effects of *R. mucronata* extracts.

Samples	AChE (mg GALAE/g)	BChE (mg GALAE/g)	Tyrosinase (mg KAE/g)	Amylase (mmol ACAE/g)	Glucosidase (mg ACAE/g)	Lipase (mg OE/g)	Elastase (mg CAE/g)
**RLM**	4.60 ± 0.02 ^a^	3.76 ± 0.13 ^b,c^	144.02 ± 0.74 ^a^	0.96 ± 0.03 ^a^	na	57.78 ± 5.00 ^e^	4.58 ± 0.04 ^a^
**RRM**	4.61 ± 0.05 ^a^	4.65 ± 0.11 ^a^	138.76 ± 1.73 ^b^	0.75 ± 0.01 ^b^	na	77.92 ± 7.36 ^d^	4.50 ± 0.16 ^a^
**RTM**	4.78 ± 0.03 ^a^	4.59 ± 0.01 ^a^	145.31 ± 1.49 ^a^	0.95 ± 0.01 ^a^	na	40.53 ± 5.06 ^c,d^	4.68 ± 0.08 ^a^
**RFM**	4.46 ± 0.18 ^a^	3.44 ± 0.05 ^b,c,d^	136.69 ± 1.22 ^b,c^	0.70 ± 0.02 ^c,d^	na	78.77 ± 9.41 ^c,d^	4.32 ± 0.11 ^a,b^
**RLD**	3.15 ± 0.10 ^d,e^	1.20 ± 0.19 ^f,g^	138.92 ± 0.15 ^b^	0.24 ± 0.01 ^g^	na	na	3.47 ± 0.38 ^c,d^
**RRD**	1.98 ± 0.31 ^f^	1.48 ± 0.50 ^e,f^	68.24 ± 0.91 ^i^	0.11 ± 0.01 ^h^	30.45 ± 1.51 ^a,b^	3.56 ± 0.88 ^g^	3.19 ± 0.21 ^d,e^
**RTD**	3.54 ± 0.01 ^c^	2.02 ± 0.66 ^e^	106.76 ± 2.00 ^f^	0.46 ± 0.08 ^f^	na	na	2.89 ± 0.37 ^e,f^
**RFD**	0.64 ± 0.13 ^h^	0.51 ± 0.05 ^g,h^	18.70 ± 0.72 ^k^	0.12 ± 0.01 ^h^	30.60 ± 0.85 ^a,b^	2.86 ± 0.28 ^g^	2.08 ± 0.49 ^h^
**RLA**	2.23 ± 0.23 ^f^	0.32 ± 0.07 ^h^	119.35 ± 0.93 ^e^	0.10 ± 0.01 ^h^	na	na	2.29 ± 0.18 ^g,h^
**RRA**	1.24 ± 0.15 ^g^	Na	44.93 ± 1.78 ^j^	0.14 ± 0.01 ^h^	31.16 ± 0.28 ^a^	na	2.55 ± 0.38 ^f,g,h^
**RTA**	3.54 ± 0.09 ^c^	2.91 ± 0.34 ^d^	100.79 ± 4.48 ^g^	0.43 ± 0.01 ^f^	na	na	3.78 ± 0.26 ^b,c^
**RFA**	0.25 ± 0.06 ^i^	na	72.14 ± 1.06 ^h^	0.12 ± 0.01 ^h^	31.16 ± 0.04 ^a^	7.44 ± 0.22 ^g^	2.67 ± 0.08 ^e,f,g^
**RLE**	3.46 ± 0.24 ^c,d^	3.34 ± 0.16 ^c,d^	132.58 ± 1.04 ^d^	0.61 ± 0.01 ^e^	29.64 ± 0.59 ^b,c^	83.10 ± 1.96 ^b,c,d^	3.65 ± 0.54 ^c,d^
**RRE**	3.64 ± 0.38 ^c^	4.15 ± 0.87 ^a,b^	131.37 ± 0.54 ^d^	0.72 ± 0.01 ^b,c^	27.61 ± 0.82 ^d^	88.32 ± 2.18 ^b^	3.47 ± 0.07 ^c,d^
**RTE**	4.04 ± 0.09 ^b^	3.62 ± 0.12 ^b,c,d^	138.94 ± 0.69 ^b^	0.73 ± 0.01 ^b,c^	30.20 ± 0.01 ^a,b^	86.14 ± 2.24 ^b,c^	3.49 ± 0.08 ^c,d^
**RFE**	2.83 ± 0.26 ^e^	4.68 ± 0.36 ^a^	134.26 ± 0.70 ^c,d^	0.66 ± 0.01 ^d^	28.77 ± 0.13 ^c^	101.02 ± 1.31 ^a^	4.25 ± 0.25 ^a^

GALAE: Galatamine equivalent; KAE: Kojic acid equivalent; ACAE: Acarbose equivalent; OE: Orlistat equivalent; CAE: Catechin equivalent; na: not active. Different letters (^a–k^) indicate significant differences in the tested extracts (*p* < 0.05). Values are expressed as means ± S.D. of three parallel measurements. Abbreviations: RLM: *Rhizophora* leaf methanolic, RRM: *Rhizophora* root methanolic, RTM: *Rhizophora* twig methanolic, RFM: *Rhizophora* fruit methanolic, RLD: *Rhizophora* leaf decoction, RRD: *Rhizophora* root decoction, RTD: *Rhizophora* twig decoction, RFD: *Rhizophora* fruit decoction, RLA: *Rhizophora* leaf aqueous RRA: *Rhizophora* root aqueous, RTA: *Rhizophora* twig aqueous, RFA: *Rhizophora* fruit aqueous, RLE: *Rhizophora* leaf ethyl acetate RRE: *Rhizophora* root ethyl acetate, RTE: *Rhizophora* twig ethyl acetate, RFE: *Rhizophora* fruit ethyl acetate.

**Table 5:** The Minimum Inhibitory Concentrations (MIC) of *R. mucronata* extracts (leaf, root, twig and fruit) against standard microorganisms.

Strains	MIC Values of *R. mucronata* Extracts (mg/mL)																
	RLM	RRM	RTM	RFM	RLE	RRE	RTE	RFE	RLA	RRA	RTA	RFA	RLD	RRD	RTD	RFD	Gentamicin (µg/mL)
*Escherichia coli* ATCC 25922	-	-	1.56	1.56	-	-	-	-	-	-	6.25	-	-	-	-	6.25	0.312
*Pseudomonas aeruginosa* ATCC 27853	-	-	-	-	-	-	-	-	6.25	-	3.13	-	6.25	-	6.25	-	0.039
*Klebsiella pneumoniae* ATCC 70603	-	-	1.56	-	-	-	-	-	3.13	-	3.13	-	-	-	6.25	-	1.25
*Staphylococcus aureus* ATCC 43300 (MRSA)	0.19	0.39	0.39	3.12	3.12	1.56	3.12	3.12	0.39	1.56	0.39	1.56	0.39	0.78	0.39	6.25	0.078
*Salmonella enteritidis* ATCC 13076	1.56	-	1.56	-	-	-	-	-	-	-	6.25	-	-	-	6.25	-	0.078
*Sarcina lutea* ATCC 9341	1.56	-	1.56	-	-	-	-	-	-	-	6.25	-	6.25	-	-	-	0.039
*Proteus mirabilis* ATCC 25933	0.39	-	0.39	-	-	-	-	-	1.56	6.25	0.78	-	1.56	6.25	1.56	-	0.312
*Bacillus cereus* ATCC 11778	-	-	-	-	-	-	-	-	-	-	3.13	-	6.25	-	-	-	<0.039
*Candida albicans* ATCC 26555	0.39	-	0.19	0.78	-	-	-	-	0.19	0.39	0.78	0.39	0.39	0.78	0.78	6.25	0.312

Abbreviations: RLM: *Rhizophora* leaf methanolic, RRM: *Rhizophora* root methanolic, RTM: *Rhizophora* twig methanolic, RFM: *Rhizophora* fruit methanolic, RLD: *Rhizophora* leaf decoction, RRD: *Rhizophora* root decoction, RTD: *Rhizophora* twig decoction, RFD: *Rhizophora* fruit decoction, RLA: *Rhizophora* leaf aqueous RRA: *Rhizophora* root aqueous, RTA: *Rhizophora* twig aqueous, RFA: *Rhizophora* fruit aqueous, RLE: *Rhizophora* leaf ethyl acetate RRE: *Rhizophora* root ethyl acetate, RTE: *Rhizophora* twig ethyl acetate, RFE: *Rhizophora* fruit ethyl acetate.

## Supplementary Materials

The following are available online at https://www.mdpi.com/2076-3921/8/10/489/s1, Table S1: dataset containing all polyphenols putatively annotated, a combined UHPLC-QTOF chromatogram, together with VIP marker compounds from OPLS-DA, when considering both organs and solvents as class membership criteria.

## Abbreviations

RLM: *Rhizophora* leaf methanolic, RRM: *Rhizophora* root methanolic, RTM: *Rhizophora* twig methanolic, RFM: *Rhizophora* fruit methanolic, RLD: *Rhizophora* leaf decoction, RRD: *Rhizophora* root decoction, RTD: *Rhizophora* twig decoction, RFD: *Rhizophora* fruit decoction, RLA: *Rhizophora* leaf aqueous RRA: *Rhizophora* root aqueous, RTA: *Rhizophora* twig aqueous, RFA: *Rhizophora* fruit aqueous, RLE: *Rhizophora* leaf ethyl acetate RRE: *Rhizophora* root ethyl acetate, RTE: *Rhizophora* twig ethyl acetate, RFE: *Rhizophora* fruit ethyl acetate.

## References

[1] Network B.O. Mangroves: Super Forests We Must Protect. https://blueocean.net/mangroves-super-forests-must-protect/.

[2] Hardoko E.S., Puspitasari Y.E., Amalia R. (2015). Study of ripe Rhizophora mucronata fruit flour as functional food for antidiabetic. Int. Food Res. J..

[3] Gurib-Fakim A., Brendler T. (2004). Medicinal and Aromatic Plants of the Indian Ocean Islands.

[4] Liebezeit G., Rau M.T. (2006). New Guinean mangroves—Traditional usage and chemistry of natural products. Senckenberg. Marit..

[5] Miles D.H., Kokpol U., Chittawong V., Tip-Pyang S., Tunsuwan K., Nguyen C. (1999). Mangrove forests—The importance of conservation as a bioresource for ecosystem diversity and utilization as a source of chemical constituents with potential medicinal and agricultural value. 1999 IUPAC.

[6] Premanathan M., Arakaki R., Izumi H., Kathiresan K., Nakano M., Yamamoto N., Nakashima H. (1999). Antiviral properties of a mangrove plant, Rhizophora apiculata Blume, against human immunodeficiency virus. Antivir. Res..

[7] Chakraborty K., Raola V.K. (2017). Two rare antioxidant and anti-inflammatory oleanenes from loop root Asiatic mangrove Rhizophora mucronata. Phytochemistry.

[8] Kusuma S., Kumar P.A., Boopalan K. (2012). Potent antimicrobial activity of Rhizophora mucronata. J. Ecobiotechnol..

[9] Adhikari A., Ray M., Das A.K., Sur T.K. (2016). Antidiabetic and antioxidant activity of Rhizophora mucronata leaves (Indian sundarban mangrove): An in vitro and in vivo study. Ayu.

[10] Sur T.K., Hazra A.K., Bhattacharyya D., Hazra A. (2015). Antiradical and antidiabetic properties of standardized extract of Sunderban mangrove Rhizophora mucronata. Pharmacogn. Mag..

[11] Aljaghthmi O., Heba H., Zeid I.A. (2018). Bioactive Compounds Extracted from Mangrove Plants (Avicennia marina and Rhizophora mucronata): An Overview. Pathophysiology.

[12] Banerjee D., Chakrabarti S., Hazra A.K., Banerjee S., Ray J., Mukherjee B. (2008). Antioxidant activity and total phenolics of some mangroves in Sundarbans. Afr. J. Biotechnol..

[13] Ramanathan T.A.H. In Studies on Analgesic Activity of a Mangrove Species—Rhizophora mucronata Poir. Proceedings of the Annual International Conference on Advances in Biotechnology (BIOTECH 2011).

[14] Sadeer N.B., Mahomoodally M.F., Zengin G., Jeewon R., Nazurally N., Rengasamy Kannan R.R., Albuquerque R.D.D.G., Pandian S.K. (2019). Ethnopharmacology, Phytochemistry, and Global Distribution of Mangroves―A Comprehensive Review. Mar. Drugs.

[15] Uysal S., Aktumsek A. (2015). A phytochemical study on Potentilla anatolica: An endemic Turkish plant. Ind. Crops Prod..

[16] Vladimir-Knežević S., Blažeković B., Bival Štefan M., Alegro A., Kőszegi T., Petrik J. (2011). Antioxidant activities and polyphenolic contents of three selected Micromeria species from Croatia. Molecules.

[17] Zengin G., Aktumsek A. (2014). Investigation of antioxidant potentials of solvent extracts from different anatomical parts of Asphodeline anatolica E. Tuzlaci: An endemic plant to Turkey. Afr. J. Tradit. Complement. Altern. Med..

[18] Rocchetti G., Giuberti G., Gallo A., Bernardi J., Marocco A., Lucini L. (2018). Effect of dietary polyphenols on the in vitro starch digestibility of pigmented maize varieties under cooking conditions. Food Res. Int..

[19] Rocchetti G., Lucini L., Rodriguez J.M.L., Barba F.J., Giuberti G. (2019). Gluten-free flours from cereals, pseudocereals and legumes: Phenolic fingerprints and in vitro antioxidant properties. Food Chem..

[20] Chlapanidas T., Faragò S., Lucconi G., Perteghella S., Galuzzi M., Mantelli M., Avanzini M.A., Tosca M.C., Marazzi M., Vigo D. (2013). Sericins exhibit ROS-scavenging, anti-tyrosinase, anti-elastase, and in vitro immunomodulatory activities. Int. J. Biol. Macromol..

[21] Grochowski D.M., Uysal S., Aktumsek A., Granica S., Zengin G., Ceylan R., Locatelli M., Tomczyk M. (2017). In vitro enzyme inhibitory properties, antioxidant activities, and phytochemical profile of Potentilla thuringiaca. Phytochem. Lett..

[22] Zengin G. (2016). A study on in vitro enzyme inhibitory properties of Asphodeline anatolica: New sources of natural inhibitors for public health problems. Ind. Crops Prod..

[23] Koc Z.E., Uysal A. (2016). Investigation of novel monopodal and dipodal oxy-Schiff base triazine from cyanuric chloride: Structural and antimicrobial studies. J. Macromol. Sci. Part A.

[24] Zengin G., Uysal A., Gunes E., Aktumsek A. (2014). Survey of phytochemical composition and biological effects of three extracts from a wild plant (Cotoneaster nummularia Fisch. et Mey.): A potential source for functional food ingredients and drug formulations. PLoS ONE.

[25] Fraga C.G., Croft K.D., Kennedy D., Tomás-Barberán F.A. (2019). The effects of polyphenols and other bioactives on human health. Food Funct..

[26] Watson R.R. (2019). Polyphenols in Plants: Isolation, Purification and Extract Preparation.

[27] Panche A.N., Diwan A.D., Chandra S.R. (2016). Flavonoids: An overview. J. Nutr. Sci..

[28] Delgoda R., Murray J.E., Badal S., Delgoda R. (2017). Chapter 7—Evolutionary Perspectives on the Role of Plant Secondary Metabolites. Pharmacognosy.

[29] Azwanida N.N. (2015). A Review on the Extraction Methods Use in Medicinal Plants, Principle, Strength and Limitation. Med. Aromat. Plants.

[30] Gololo S.S. (2018). Potential Adverse Effects of Alteration of Phytochemical Accumulation in Fruits and Vegetables. Phytochemicals—Source of Antioxidants and Role in Disease Prevention.

[31] Iriti M., Faoro F. (2009). Chemical Diversity and Defence Metabolism: How Plants Cope with Pathogens and Ozone Pollution. Int. J. Mol. Sci..

[32] Suganthy N., Pandima Devi K. (2016). In vitro antioxidant and anti-cholinesterase activities of Rhizophora mucronata. Pharm. Biol..

[33] Dutta S., Ray S. (2018). Comparative assessment of total phenolic content and in vitro antioxidant activities of bark and leaf methanolic extracts of Manilkara hexandra (Roxb.) Dubard. J. King Saud Univ. Sci..

[34] Carocho M., Ferreira I.C.F.R. (2013). A review on antioxidants, prooxidants and related controversy: Natural and synthetic compounds, screening and analysis methodologies and future perspectives. Food Chem. Toxicol..

[35] Kim D.O., Lee K.W., Lee H.J., Lee C.Y. (2002). Vitamin C Equivalent Antioxidant Capacity (VCEAC) of Phenolic Phytochemicals. J. Agric. Food Chem..

[36] Cheng Z., Li Y. (2004). Reducing power: The measure of antioxidant activities of reductant compounds?. Redox Rep..

[37] Tanaka M., Kuei C.W., Nagashima Y., Taguchi T. (1988). Application of Antioxidative Maillard Reaction Products from Histidine and Glucose to Sardine Products. Nippon Suisan Gakkaishi.

[38] Segura Campos M.R., Ruiz Ruiz J., Chel-Guerrero L., Betancur Ancona D. (2015). Coccoloba uvifera (L.) (Polygonaceae) Fruit: Phytochemical Screening and Potential Antioxidant Activity. J. Chem..

[39] Abdel-Hameed E.S.S., Bazaid S.A., Salman M.S. (2013). Characterization of the Phytochemical Constituents of Taif Rose and Its Antioxidant and Anticancer Activities. BioMed Res. Int..

[40] Schmidt C. (2018). The fight against non-communicable disease in emerging economies. Nature.

[41] Tucci S.A., Boyland E.J., Halford J.C. (2010). The role of lipid and carbohydrate digestive enzyme inhibitors in the management of obesity: A review of current and emerging therapeutic agents. Diabetes Metab. Syndr. Obes..

[42] Purnell J., Kopelman P.G., Caterson I.D., Dietz W.H. (2018). Definitions, Classification, and Epidemiology of Obesity. Clinical Obesity in Adults and Children.

[43] WHO Obesity. https://www.who.int/topics/obesity/en/.

[44] Pearson H. (2003). In the eye of the beholder. Nature.

[45] Stern J.S., Kazaks A. (2015). Obesity: A Reference Handbook.

[46] Ramirez G., Zavala M., Perez J., Zamilpa A. (2012). In Vitro Screening of Medicinal Plants Used in Mexico as Antidiabetics with Glucosidase and Lipase Inhibitory Activities. Evid.-Based Complement. Altern. Med..

[47] Bustanji Y., Mohammad M., Hudaib M., Tawaha K., Almasri I., Alkhatib H., Issa A., Alali F. (2011). Screening of some medicinal plants for their pancreatic lipase inhibitory potential. Jordan J. Pharm. Sci..

[48] Nature Neurodegenerative Diseases. https://www.nature.com/subjects/neurodegenerative-diseases.

[49] Bilska A., Kobus-Cisowska J., Kmiecik D., Danyluk B., Kowalski R., Szymanowska D., Gramza-Michałowska A., Szczepaniak O. (2019). Cholinesterase inhibitory activity, antioxidative potential and microbial stability of innovative liver pâté fortified with rosemary extract (Rosmarinus officinalis). Electron. J. Biotechnol..

[50] Williams P., Sorribas A., Howes M.J.R. (2011). Natural products as a source of Alzheimer’s drug leads. Nat. Prod. Rep..

[51] Dorni C.A.I., Amalraj A., Gopi S., Varma K., Anjana S.N. (2017). Novel cosmeceuticals from plants—An industry guided review. J. Appl. Res. Med. Aromat. Plants.

[52] Brozyna A., Zbytek B., Granese J., Carlson J.A., Ross J., Slominski A. (2007). Mechanism of UV-related carcinogenesis and its contribution to nevi/melanoma. Expert Rev. Dermatol..

[53] Mapunya M.B., Nikolova R.V., Lall N. (2012). Melanogenesis and Antityrosinase Activity of Selected South African Plants. Evid.-Based Complement. Altern. Med..

[54] Butt A.R.S., Abbasi M.A., Aziz Ur R., Siddiqui S.Z., Hassan M., Raza H., Shah S.A.A., Seo S.Y. (2019). Synthesis and structure-activity relationship of elastase inhibiting novel ethylated thiazole-triazole acetamide hybrids: Mechanistic insights through kinetics and computational contemplations. Bioorgan. Chem..

[55] Tomasz A. (1994). Multiple-Antibiotic-Resistant Pathogenic Bacteria—A Report on the Rockefeller University Workshop. N. Engl. J. Med..

[56] Klevens R.M., Morrison M.A., Nadle J., Petit S., Gershman K., Ray S., Harrison L.H., Lynfield R., Dumyati G., Townes J.M. (2007). Invasive methicillin-resistant Staphylococcus aureus infections in the United States. Jama.

[57] Centers for Disease Control and Prevention (1997). Staphylococcus aureus with reduced susceptibility to vancomycin—United States, 1997. MMWR. Morb. Mortal. Wkly. Rep..

[58] Hiramatsu K., Hanaki H., Ino T., Yabuta K., Oguri T., Tenover F. (1997). Methicillin-resistant Staphylococcus aureus clinical strain with reduced vancomycin susceptibility. J. Antimicrob. Chemother..

